# The Two Translationally Controlled Tumor Protein Genes, *CsTCTP1* and *CsTCTP2*, Are Negative Modulators in the *Cucumis sativus* Defense Response to *Sphaerotheca fuliginea*

**DOI:** 10.3389/fpls.2018.00544

**Published:** 2018-04-25

**Authors:** Xiangnan Meng, Yang Yu, Junyue Zhao, Na Cui, Tiefeng Song, Yun Yang, Haiyan Fan

**Affiliations:** ^1^College of Bioscience and Biotechnology, Shenyang Agricultural University, Shenyang, China; ^2^College of Horticulture, Shenyang Agricultural University, Shenyang, China; ^3^Vegetable Research Institute, Liaoning Academy of Agricultural Sciences, Shenyang, China; ^4^Key Laboratory of Protected Horticulture of Ministry of Education, Shenyang Agricultural University, Shenyang, China

**Keywords:** ABA signaling pathway, *Cucumis sativus*, Sphaerotheca fuliginea, translationally controlled tumor protein, transient transformation, TOR signaling pathway

## Abstract

Pathogen stress often significantly decreases cucumber production. However, knowledge regarding the molecular mechanism and signals of cucumber disease resistance is far from complete. Here, we report two translationally controlled tumor protein genes, *CsTCTP1* and *CsTCTP2*, that are both negative modulators in the *Cucumis sativus* defense response to *Sphaerotheca fuliginea*. Subcellular localization analysis showed that CsTCTP1 and CsTCTP2 were both localized in the cytoplasm. Expression analysis indicated that the transcript levels of *CsTCTP1* and *CsTCTP2* were linked to the degree of cucumber resistance to *S. fuliginea*. Transient overexpression of either *CsTCTP1* or *CsTCTP2* in cucumber cotyledons impaired resistance to *S. fuliginea*, whereas silencing of either *CsTCTP1* or *CsTCTP2* enhanced cucumber resistance to *S. fuliginea.* The relationship of several defense-related genes and ABA and target of rapamycin (TOR) signaling pathway-related genes to the overexpressing and silencing of *CsTCTP1*/*CsTCTP2* in non-infested cucumber plants was investigated. The results indicated that *CsTCTP1* participates in the defense response to *S. fuliginea* by regulating the expression of certain defense-associated genes and/or ABA signaling pathway-associated genes, and *CsTCTP2* participates through regulating the expression of TOR signaling pathway-associated genes. Our findings will guide enhancing the resistance of cucumber to powdery mildew.

## Introduction

Powdery mildew (PM) caused by *Sphaerotheca fuliginea* occurs worldwide and is one of the most destructive cucumber diseases in many mild and damp regions (Schulze-Lefert and Vogel, 2000). PM fungi are biotrophic parasites that spread through the air and greatly depress yields worldwide. After a conidial spore absorbs on the host surface, the PM fungus builds one or two germ tubes. An appressorial germ tube develops an appressorium and then penetrates the host cell wall (Hückelhoven, 2005). After the first barrier (the host cell wall) is defeated, the PM fungus develops a haustorium and takes nutrients from the host. A hypha simultaneously continues to spread on the host epidermis. The host defense that inhibits nutrient uptake is thus considered the second barrier.

Increasing amounts of plant data indicate that similar to other eukaryotic phyla, the target of rapamycin (TOR) acts as a master regulator of nutrient, hormone and stress signaling networks (Robaglia et al., 2012; Xiong and Sheen, 2015). Unlike the TOR kinases of animals and yeasts, the plant TOR kinase is present in a TORC1 protein complex, which consists of the TOR, the regulatory-associated protein of TOR (Raptor) and lethal with SEC13 protein 8 (LST8) (Anderson et al., 2005; Laplante and Sabatini, 2012; Moreau et al., 2012; Maegawa et al., 2015). TOR is a conserved Ser/Thr protein kinase, and its N-terminal region interacts with Raptor WD repeats (Adami et al., 2007). Raptor may be involved in substrate recruitment for phosphorylation by TOR, and LST8 might stabilize the complex (Hara et al., 2002; Turck et al., 2004). Evidence also suggests that possible TOR substrates, such as ribosome protein S6 kinase (S6K) and the type 2A-phosphatase-associated protein of 46 kDa (TAP46, a regulator of PP2A phosphatases), are well conserved in plants (Nojima et al., 2003; Ahn et al., 2014). In *Arabidopsis*, TOR signaling is inhibited upon the interaction of AtRaptor1 and the AtS6K1 N-terminal 44-amino acid region (Son et al., 2016). Cucumber only harbors one *S6K* gene, and this gene is very similar to *AtS6K1* and *AtS6K2* (approximately 70% identity; Pu et al., 2017). In animals, TORC1 is inactivated upon phosphorylation of the Raptor protein by AMP-activated kinase (AMPK). Sucrose non-fermenting 1-related kinase 1 (SnRK1), the plant homolog of AMPK, is deduced to have similar functions in inactivating plant TORC1 (Gwinn et al., 2008; Dobrenel et al., 2016). Under stress conditions, SnRK1 is activated to suppress energy-demanding processes, which are positive regulated by TOR, and activate stress-dependent energy-generating processes, thus maintaining energy homeostasis (Baena-González and Sheen, 2008; Jossier et al., 2009).

Plants can escape from pathogen assault owing to a plethora of factors. The expression of defense-associated genes is also an important part in plant–pathogen interactions. For instance, *pathogenesis-related protein-1a* (*PR-1a*) and *14-3-3* genes are also thought to be involved in the response of cucumber plants to *S. fuliginea* (Liu, 2008; Zhang, 2016). Additionally, chitinase, a secondary hydrolytic enzyme, can inhibit hyphal growth by hydrolyzing chitin in the cell wall of mycelial growth tips and thus display resistance against fungal pathogens in plants (Luo et al., 2015). The cucumber pathogen-induced protein CuPi1, a phloem lectin protein, was more abundant in response to *S. fuliginea* invasion in susceptible cucumber lines than resistant lines (Meng et al., 2016).

The translationally controlled tumor protein (TCTP) is a highly conserved multifunctional protein in Eukarya. Published data revealed that animal TCTP is implicated in an amazing number of cellular events, including cell growth, differentiation, development of organ size, apoptosis, signaling, and stimulus and immune responses (Yang et al., 2005; Hsu et al., 2007; Schmidt et al., 2007; Susini et al., 2008; Vonakis et al., 2008). Great progress has been made in understanding TCTP functions in animals and yeasts, but knowledge of this gene in plants has lagged. The *Arabidopsis* TCTP gene rescues the corresponding *Drosophila* TCTP mutant (Brioudes et al., 2010), which underscores the similarity of its function with those of plant and non-plant homologs. Emerging evidence suggests a role of plant TCTP in growth regulation and programmed cell death control (Berkowitz et al., 2008; Toscano-morales et al., 2015; Chou et al., 2016). Plant TCTP was also recently found to respond to pathogen stress (Li et al., 2010; Hinojosamoya et al., 2013; Bruckner et al., 2017). In our previous work, we found that TCTP (XP-004134215) was significantly differentially expressed between two sister cucumber lines (genotypes that were either highly resistant or highly susceptible to *S. fuliginea*) by 2-D gel electrophoresis (Fan et al., 2014). Cucumber has 2 *TCTP* genes, *CsTCTP1* (XP-004134215) and *CsTCTP2* (XP-004135602). Though CsTCTP1 and CsTCTP2 share a high degree of homology, the functions of CsTCTPs to different abiotic stresses may differ (Meng et al., 2017). The molecular and biochemical mechanisms by which TCTP functions in response to *S. fuliginea* have not been completely established.

TCTP, which is similar to members of the mammalian/dominant suppressor of sec4 (MSS4/DSS4) protein family, contains a domain that putatively binds Rab GTPase. TCTP is also a vital component of the TOR signaling network (Berkowitz et al., 2008; John et al., 2011). Indeed, *Arabidopsis* TCTP can bind to four AtRab GTPases (AtRABA4a, AtRABA4b, AtRABF1, and AtRABF2b) and interact with Drosophila dRheb GTPases (Brioudes et al., 2010). Additionally, the interaction between TCTP and Rheb is regulated by 14-3-3 (Le et al., 2016). However, the specific relationships between CsTCTP and the TOR signaling pathway in response to *S. fuliginea* remain unknown.

*CsTCTP1* and *CsTCTP2* are both regulated by exogenous abscisic acid (ABA) treatment (Meng et al., 2017). Moreover, *AtTCTP* overexpression enhanced drought tolerance in *Arabidopsis* and played a role in ABA-mediated stomatal movement (Kim et al., 2012). Accumulating evidence indicates that plant TCTP is related to ABA signal transduction. ABA is also a critical signaling molecule that mediates plant defense responses against pathogen stress (Cao et al., 2011). The ABA signal transduction core components of plants contain ABA receptors (PYR/PYL/RCARs), group A type 2C protein phosphatases (PP2Cs) and subclass III SNF 1-related protein kinases 2 (SnRK2s). The ABA-insensitive (ABI5) gene is thought to be a downstream target of the ABA signaling pathway. In cucumbers, PYL2, PP2C2, and SnRK2.2 are reportedly involved in transducing ABA signals (Wang et al., 2012). The specific relationships between CsTCTP and the TOR signaling pathway in response to *S. fuliginea* are similarly poorly understood.

In this study, the subcellular localization and expression patterns of *CsTCTP1* and *CsTCTP2* were determined. Investigation of their defense roles via transient *CsTCTP1*/*CsTCTP2*-overexpression and *CsTCTP1*/*CsTCTP2*-silencing cucumber plants elucidated that CsTCTP1 and CsTCTP2 both function as negative regulators in host resistance response to *S. fuliginea*. Furthermore, *CsTCTP1* might participate in the defense response to *S. fuliginea* through regulating the expression of certain defense-associated genes and/or ABA signaling pathway-associated genes, and *CsTCTP2* may act by regulating the expression of TOR signaling pathway-associated genes.

## Materials and Methods

### Plant Materials and Treatments

The cucumber lines/cultivars used in this study were B21-a-2-2-2, XinTaiMiCi, JingYan4, GY14, and B21-a-2-1-2. B21-a-2-2-2 (highly susceptible to *S. fuliginea*) and B21-a-2-1-2 (highly resistant to *S. fuliginea*) are two sister cucumber lines that were obtained from the Liaoning Academy of Agricultural Sciences. XinTaiMiCi is susceptible to *S. fuliginea* and was selected for gene transformation. GY14, an important processing cultivar in North America, is provided by Shanghai Jiao Tong University. JingYan4 is an important planting variety in China that is resistant to downy mildew, PM, and so on.

Cucumber plants were grown in a growth chamber with day/night temperatures of 25/22°C and a 16-h light photoperiod. For rapamycin treatment, XinTaiMiCi cucumbers were grown in a liquid culture to which 10 μM rapamycin had been added. Seven-day-old seedlings of five cucumber lines/cultivars were inoculated with *S. fuliginea* by spore spray as described previously (Ren, 2011). The cotyledons collected at day 7 of *S. fuliginea* treatment were placed in liquid nitrogen and stored at -80°C until future study.

### Subcellular Localization

The full-length *CsTCTP1* and *CsTCTP2* ORFs without their stop codons were separately amplified by PCRs and ligated to the 3′ end of the green fluorescent protein (GFP) coding region, yielding p35S:GFP-*CsTCTP1* and p35S:GFP-*CsTCTP2*. The resulting p35S:GFP-*CsTCTP1*, p35S:GFP-*CsTCTP2*, and control p35S:GFP plasmids were separately injected into *Nicotiana benthamiana* leaves and *Arabidopsis thaliana* protoplasts following the approach of Zhang et al. (2017) and Yoo et al. (2007). The plants were incubated at 22°C for 48 h after injection, at which point GFP signals were observed and photographed using a laser confocal florescence microscope (Leica, TSC SP8, Germany) with an excitation wavelength of 488 nm and a 505–530 nm bandpass emission filter.

### TRV-Mediated *CsTCTP1* and *CsTCTP2* Gene Silencing in Cucumber Cotyledons

A 318 bp sequence of *CsTCTP1* (from nucleotides 1 to 318 in the *CsTCTP1* cDNA sequence) or a 318 bp sequence of *CsTCTP2* (from nucleotides 1 to 318 in the *CsTCTP2* cDNA sequence) was subcloned in an antisense orientation into the plasmid pTRV2, yielding pTRV2-*CsTCTP1* and pTRV2-*CsTCTP2*, respectively, which were then introduced into different *Agrobacterium tumefaciens* EHA105 aliquots. Cotyledons of 8-day-old cucumber seedlings were injected with a mixture (1:1, ratio, v/v) of *Agrobacterium* cultures containing pTRV1 and a pTRV2, pTRV2-*CsTCTP1*, or pTRV2-*CsTCTP2* plasmid using a needleless syringe. The cotyledons at 7 days post-injection with TRV:00, TRV:*CsTCTP1*, or TRV:*CsTCTP2* were photographed to record their phenotypes. Cotyledons at 14 days post-injection were collected for RNA extraction and molecular detection experiments. Cotyledons at 7 days post-injection were used for *S. fuliginea* inoculation and fungal biomass detection.

### *CsTCTP1* and *CsTCTP2* Overexpression Transformation Vector and Cucumber Transient Transformation

The cDNAs of *CsTCTP1* and *CsTCTP2* were subcloned into separate pCAMBIA3301 binary vectors modified with *Xba* I and *Pst* I, where they were placed under the control of the 35S promoter. The vector contains a luciferase reporter gene. LUC:00, LUC:*CsTCTP1*, and LUC:*CsTCTP2* vectors were individually transferred into *A. tumefaciens* strain EHA105.

*Agrobacterium tumefaciens* EHA105 cells harboring the LUC:00, LUC:*CsTCTP1*, or LUC:*CsTCTP2* vector were cultured to OD600 = 0.8 in induction medium (10 mM MES, 10 mM MgCl_2_, and 200 mM acetosyringone) and then diluted to OD600 = 0.4. The diluted culture was injected into 8-day-old cucumber cotyledons using a syringe without a needle (Shang et al., 2014). For luciferase assays, 7-day post-injection cotyledons were punched into 1.5-cm diameter round pieces, soaked in 0.3125 mg mL^-1^ D-luciferin for 45 min and then photographed by an *in vivo* Plant Imaging System (Berthold, Lb985). For molecular detection experiments, cotyledons with LUC:00, LUC:*CsTCTP1*, or LUC:*CsTCTP2* were collected at 5 days post-injection, placed in liquid nitrogen and stored at -80°C until further studied. Cotyledons with LUC:00, LUC:*CsTCTP1* or LUC:*CsTCTP2* at 1 day post-injection were used for *S. fuliginea* treatment for fungal biomass detection.

### PCR and qRT-PCR Analysis

Total RNA was isolated using an RNAprep pure Plant Kit(DP432, TIANGEN, China), and cDNA was synthesized by a QuantScript RT Kit (KR103-04, TIANGEN, China). The presence of the introduced *CsTCTP1* or *CsTCTP2* gene in the transformed cucumber plants was detected by PCRs using a specific primer pair. The two forward primers were CsTCTP1-F, targeting part of the *CsTCTP1* gene region, and CsTCTP2-F, targeting part of the *CsTCTP2* gene region, and the reverse primer for both cases was LUC-R, targeting part of the luciferase sequence region of the transformation vector. PCRs were conducted in a 20 μL volume containing 1 μL of genomic DNA, 2 μL of Taq DNA Polymerase (TIANGEN), 2 μL of 10 × Taq buffer, 0.5 μL of each primer (10 μM), and 12.5 μL of doubly distilled water.

The relative expression levels of *CsTCTP1, CsTCTP2*, defense-related genes, and TOR- and ABA-signaling-related genes were analyzed by qRT-PCR. The qRT-PCRs were performed on a SYBR Green I 96-I system (Roche fluorescence quantitative PCR instrument, Basel). The cucumber *actin* gene, *CsActin*, was used as an internal reference. The gene-specific primers that were employed are shown in Supplementary Table S1. The expression patterns of the target genes were calculated using the 2^-ΔΔC_T_^ method. Three independent biological replicates and three technical replicates for each biological replicate were performed, and significance was determined by *t*-test using SPSS statistical software (*P* < 0.05).

### Coomassie Brilliant Blue R250 Staining and Microscopy

Coomassie brilliant blue R250 staining was used for observing the *S. fuliginea* hyphae on the cotyledons of control, LUC:00, LUC:*CsTCTP1*, LUC:*CsTCTP2*, TRV:00, TRV:*CsTCTP1*, and TRV:*CsTCTP2* transgenic plants at day 7 with *S. fuliginea*. Control and transgenic infected cotyledons were immersed in destaining solution (containing 22.5 mg of trichloroacetic acid, 15 mL of absolute ethanol, and 5 mL of trichloromethane) for 1 h at 70°C. The samples were then stained with Coomassie brilliant blue R250 solution (containing 3 mg of trichloroacetic acid, 20 mL of H_2_O, 120 mg of Coomassie brilliant blue R250, and 20 mL of methanol) for 15 min at room temperature. After the samples had been washed with sterile water, they were immersed in 20% glycerol and photographed. The fungus was visualized under a light microscope (Nikon).

### Disease Index

The typical symptoms of pathogen whiteness were measured in six disease severity ratings from 0 to 9, where 0 = no symptoms, 1 = 5% of inoculated leaves were whitish, 3 = 5–30% of inoculated leaves were whitish, 5 = 30–50% of inoculated leaves were whitish, 7 = 50–75% of inoculated leaves were whitish, 9 = 75–100% of the inoculated leaves were whitish, and plant death. A Disease Index (DI) was calculated using the following formula: DI (%) = [Σ (*N* × *D*)/(*H* × *T*)] × 100, where *N* = number of plants with the respective disease rating; *D* = the disease rating; *H* = the highest disease rating; and *T* = the total number of observed plants. No fewer than 30 cucumbers were surveyed for DI evaluation.

### Statistical Analysis

Primer design and sequence alignment were conducted in Primer 5 software. Standard errors of deviation were assessed by Excel. Statistical significance was analyzed by Student’s *t*-test (*P* < 0.05) using SPSS software.

## Results

### Molecular Characterization and Expression Patterns of *CsTCTP1* and *CsTCTP2*

**FIGURE 1:**
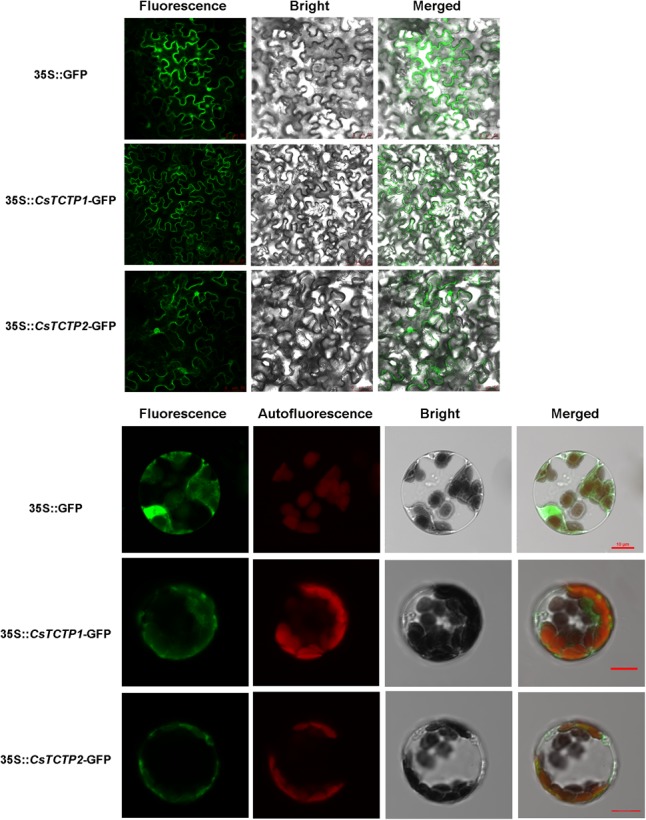
Subcellular localization of GFP:*CsTCTP1* and GFP:*CsTCTP2* fusion proteins in *N. benthamiana* cells and *Arabidopsis* protoplasts. Fluorescence, chloroplast autofluorescence, bright field, and merged image were obtained using a Leica confocal microscope.

Cucumber harbors two TCTP paralogs with 77% nucleic acid identity, *CsTCTP1* (XP-004134215), and *CsTCTP2* (XP-004135602), both of which encoded 168 amino acids. Sequence analysis showed that both CsTCTP1 and CsTCTP2 proteins contained conserved TCTP motifs, such as a domain that putatively binds Rab GTPase and TCTP1 (45–55) and TCTP2 (125–147) domains. To explore the subcellular localization of CsTCTP1 and CsTCTP2, we separately infiltrated and transiently transformed p35S:GFP-*CsTCTP1*, p35S:GFP-*CsTCTP2*, and control p35S:GFP vectors into *N. benthamiana* leaves and *A. thaliana* protoplasts. Confocal microscope examination indicated the exclusive localization of p35S:GFP-*CsTCTP1* and p35S:GFP-*CsTCTP2* in the cytoplasm, whereas only GFP alone was found in multiple subcellular compartments, including the cytoplasm and nucleus (**Figure 1**). The results are in accordance with *in silico* analysis data predicting that CsTCTP1 and CsTCTP2 are both localized in cytoplasm (Chen, 2017).

The patterns of *CsTCTP1* and *CsTCTP2* expression were investigated in five cucumber lines with different degrees of resistance, including the highly susceptible line B21-a-2-2-2, the susceptible line XinTaiMiCi, the moderately resistant lines JinYan 4 and GY14, and the highly resistant line B21-a-2-1-2, with or without *S. fuliginea* inoculation for 7 days (Supplementary Figure S1). Without *S. fuliginea* inoculation, the expression levels of *CsTCTP1* and *CsTCTP2* were not significantly different in the five cucumber lines. With *S. fuliginea* infestation, *CsTCTP1* and *CsTCTP2* transcription increased in susceptible lines and declined in resistant lines. The maximum expression of *CsTCTP1* and *CsTCTP2* occurred in the B21-a-2-2-2 line with *S. fuliginea* treatment. The results demonstrated that the different expression patterns of *CsTCTP1* and *CsTCTP2* in *S. fuliginea*-susceptible and *S. fuliginea*-resistant plants after pathogen inoculation are closely linked to the magnitude of cucumber resistance to *S. fuliginea* and that *CsTCTP1* and *CsTCTP2* may be involved in the cucumber defense response to *S. fuliginea*.

### *CsTCTP1* or *CsTCTP2* Silencing Improves Resistance to *S. fuliginea* in Transgenic Cucumber

**FIGURE 2:**
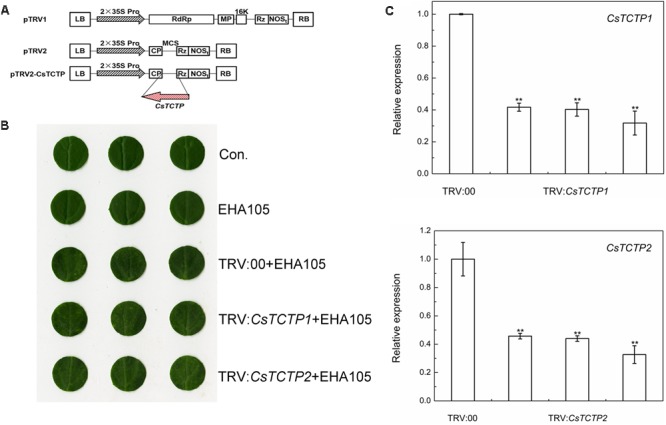
Identification of *CsTCTP*-silenced cucumber plants. **(A)** *CsTCTP1-* and *CsTCTP2*-silencing transformation vectors pTRV-*CsTCTP1* and pTRV-*CsTCTP2*. The arrows show the direction of the inserted fragments. **(B)** Symptoms in detached cotyledons of silenced cucumber plants. Con. indicates non-injected plants, and EHA105 indicates EHA105-injected plants. **(C)** qRT-PCR analyses of the expression levels of *CsTCTP1* in cucumber plants injected with TRV:00 or TRV:*CsTCTP1* and the expression levels of *CsTCTP2* in cucumber plants injected with TRV:00 or TRV:*CsTCTP2*. Statistically significant differences were identified based on three technical replicates (*t*-test; ^∗^*P* < 0.05, ^∗∗^*P* < 0.01). Bars indicate the standard error of the mean values, and data are presented as the mean value ± SE of three biological replicates.

To elucidate whether *CsTCTP1* or *CsTCTP2* was required for cucumber resistance to *S. fuliginea*, we used tobacco rattle virus (TRV)-based virus-induced gene silencing (VIGS) to silence *CsTCTP1*/*CsTCTP2* transcripts in the susceptible line XinTaiMiCi. A 5′-terminal fragment specific to *CsTCTP1*/*CsTCTP2* was inserted in an antisense orientation into vector pTRV2, yielding the pTRV2-*CsTCTP1*/*CsTCTP2* recombinant plasmid (**Figure 2A**). When either the TRV:*CsTCTP1* or TRV:*CsTCTP2* virus was injected with TRV:00 for 7 days, the chlorotic mosaic symptoms of TRV emerged in the cotyledons of transgenic cucumber plants; however, no symptoms appeared in the control and EHA105-injected cucumbers, suggesting that TRV successfully invaded the cucumber plants injected with TRV:00 and either TRV:*CsTCTP1* or TRV:*CsTCTP2* (**Figure 2B**). The transcript levels of *CsTCTP1* in TRV:*CsTCTP1* transgenic plants and *CsTCTP2* in TRV:*CsTCTP2* transgenic plants were markedly lower than those of *CsTCTP1* and *CsTCTP2*, respectively, in TRV:00-injected plants (**Figure 2C**). These results indicated that *CsTCTP1*/*CsTCTP2*-silenced plants were successfully generated.

**FIGURE 3:**
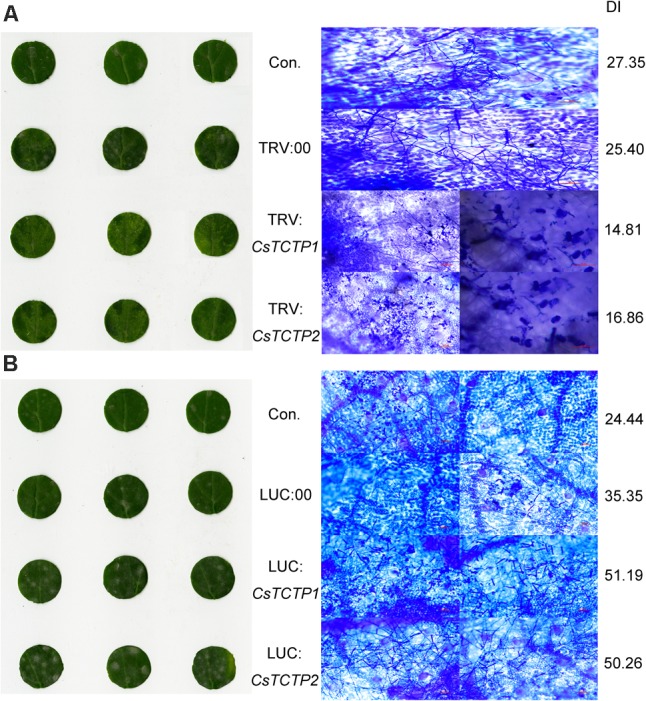
Silencing of either *CsTCTP1* or *CsTCTP2* enhanced cucumber resistance to *S. fuliginea*, whereas transient overexpression of either *CsTCTP1* or *CsTCTP2* in cucumber cotyledons impaired resistance to *S. fuliginea.* **(A)** Disease symptoms of detached cotyledons of control and *CsTCTP1*/*CsTCTP2*-silenced transgenic cucumbers at 7 dpi with *S. fuliginea*, and Coomassie brilliant blue staining for the examination of the *S. fuliginea* hyphae of cotyledons of control and *CsTCTP1*/*CsTCTP2*-silenced transgenic cucumbers at 7 dpi with *S. fuliginea.* **(B)** Disease symptoms of detached cotyledons of control and *CsTCTP1*/*CsTCTP2*-overexpressed transgenic cucumbers at 7 dpi with *S. fuliginea*, and Coomassie brilliant blue staining for the examination of the *S. fuliginea* hyphae of cotyledons of control and *CsTCTP1*/*CsTCTP2*-overexpressed transgenic cucumbers at 7 dpi with *S. fuliginea.* DI indicates the disease index of PM.

These *CsTCTP1*-silenced cucumbers and *CsTCTP2*-silenced cucumbers both exhibited significantly enhanced resistance to *S. fuliginea* after 7 days of infection (**Figure 3A**). Many hyphae and branch spores invaded control and TRV:00-injected plants, but only a few initial germ tubes and appressoria were observed in *CsTCTP1/CsTCTP2*-silenced cucumbers, suggesting that *CsTCTP1/CsTCTP2*-silencing increases resistance to hyphal development of *S. fuliginea* (**Figure 3A**). Furthermore, the DI of the *CsTCTP1-*silenced plants that had been inoculated with *S. fuliginea* was 14.81, and the DI of the inoculated *CsTCTP2*-silenced plants was 16.86, whereas the DIs of the control and TRV:00-injected plants were 27.35 and 25.40, respectively (**Figure 3A**). These results indicated that the silencing of *CsTCTP1* or *CsTCTP2* enhanced plant resistance to *S. fuliginea.*

### Transient Overexpression of *CsTCTP1* or *CsTCTP2* in Cucumber Cotyledons Impairs Resistance to *S. fuliginea*

**FIGURE 4:**
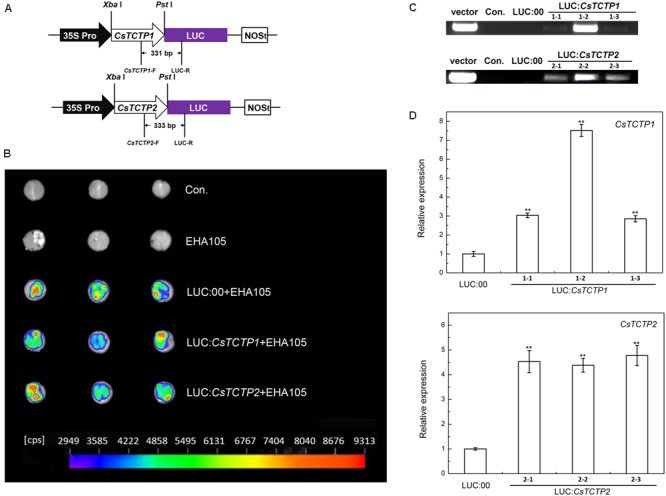
Identification of *CsTCTP*-overexpressing cucumber plants. **(A)** *CsTCTP1-* and *CsTCTP2*-overexpressing transformation vectors LUC:*CsTCTP1* and LUC:*CsTCTP2*. The arrows show the fragment that was amplified in the PCR detection of the recombinant plasmids. **(B)** Luminescence of detached cotyledons of transient overexpression cucumber plants. Con. indicates non-injected plants, EHA105 indicates EHA105-injected plants, and indicates the number of counts per second. **(C)** PCR identification of LUC:*CsTCTP1*-injected and LUC:00-injected cucumbers using primers specific to the CsTCTP1-LUC cassette, and PCR identification of LUC:*CsTCTP2*-injected and LUC:00-injected cucumbers using primers specific to the CsTCTP2-LUC cassette. Con. indicates non-injected cucumber plants, and vector indicates the transformation vector. **(D)** qRT-PCR analyses of the expression levels of the *CsTCTP1* gene in cucumber plants injected with LUC:00 or LUC:*CsTCTP1* and the expression levels of the *CsTCTP2* gene in cucumber plants injected with LUC:00 or LUC:*CsTCTP2*. Statistically significant differences were identified based on three technical replicates (*t*-test; ^∗^*P* < 0.05, ^∗∗^*P* < 0.01). Bars indicate the standard error of the mean values, and data are presented as the mean values ± SE of three biological replicates.

To gain further insights into the response of *CsTCTP1/CsTCTP2* function to *S. fuliginea*, we constructed the overexpression transformation plasmids LUC:*CsTCTP1* and LUC:*CsTCTP2*, and LUC:00 (**Figure 4A**), LUC:*CsTCTP1* and LUC:*CsTCTP2* were separately transformed into and transiently expressed in cucumber XinTaiMiCi cotyledons. Luminescence indicated the expression of *LUC* in the LUC:00-, LUC:*CsTCTP1*-, and LUC:*CsTCTP2*-injected cucumber plants, but the non-injected (control) and EHA105-injected plants did not luminesce (**Figure 4B**). PCR analysis using the primers CsTCTP1-F/CsTCTP2-F and LUC-R showed that the introduced transgene was only found in *CsTCTP1*/*CsTCTP2*-overexpressing plants (**Figure 4C**). Then, qRT-PCR results showed that the transcript levels of *CsTCTP1* in LUC:*CsTCTP1* and *CsTCTP2* in LUC:*CsTCTP2* transgenic cucumbers were markedly higher than those of LUC:00-injected plants (**Figure 4D**). These results indicated that *CsTCTP1* and *CsTCTP2* genes had been successfully transiently overexpressed in the transgenic plants.

At day 6 with *S. fuliginea*, the cotyledons of *CsTCTP1*/-*CsTCTP2*-overexpressing plants exhibited the whitish appearance typical of PM disease, but control and LUC:00-injected plants demonstrated this appearance to a lesser extent. Microscope observations showed that the hyphal abundances of *S. fuliginea* were greater on the infested leaves of the *CsTCTP1*/*CsTCTP2*-overexpressing plants than on those of control and LUC:00-injected plants. Moreover, the DI of *CsTCTP1*-overexpressing plants was 51.19, and the DI of *CsTCTP2*-overexpressing plants was 50.26, while the DIs of the control and LUC:00-injected plants were 24.44 and 35.35, respectively (**Figure 3B**). These results suggested that overexpression of *CsTCTP1* or *CsTCTP2* in XinTaiMiCi plants impaired their resistance to *S. fuliginea* and that *CsTCTP1* and *CsTCTP2* are required for the cucumber defense response to *S. fuliginea* infections.

### *CsTCTP1* and *CsTCTP2* Transgenic Plants Modulate the Expression Levels of Defense-Associated Genes

**FIGURE 5:**
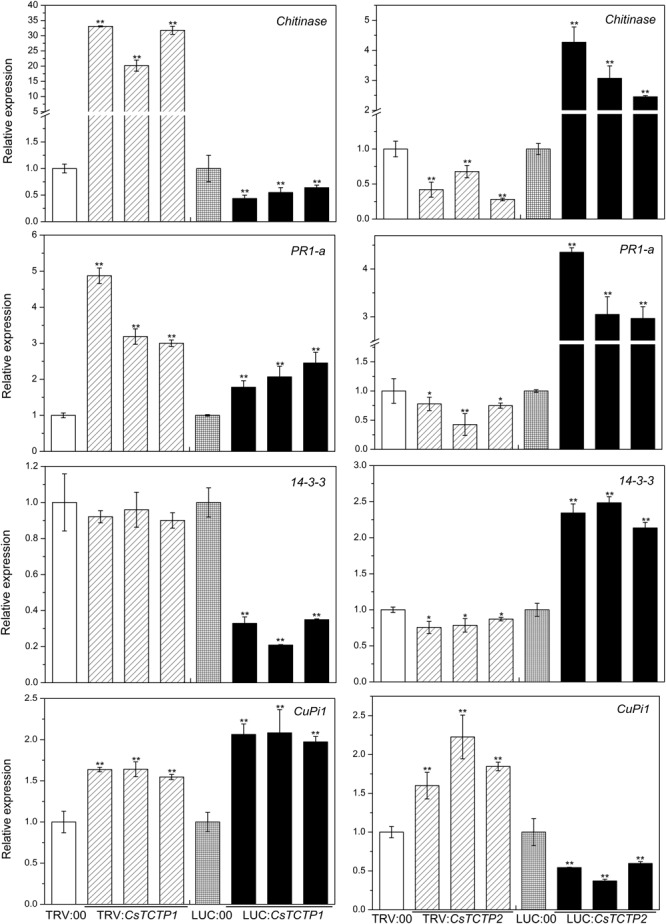
Relative expression levels of four genes (*chitinase, PR-1a, 14-3-3*, and *CuPi1*) related to *S. fuliginea* resistance in cucumber. The reported transcription levels of the tested genes in the *CsTCTP1*/*CsTCTP2*-silenced and *CsTCTP1*/*CsTCTP2*-overexpressing plants are relative to those in the TRV:00- and LUC:00-injected plants, respectively. Statistically significant differences were identified based on three technical replicates (*t*-test; ^∗^*P* < 0.05, ^∗∗^*P* < 0.01). Bars indicate the standard error of the mean, and data are presented as the mean values ± SE of three biological replicates.

To elucidate whether *CsTCTP1*/*CsTCTP2*-mediated resistance against *S. fuliginea* is related to pathogen-associated genes, we examined the transcriptional responses of four known *S. fuliginea*-related defense genes, including *14-3-3* (XM-004136782.2), *chitinase* (HM015248), *PR-1a* (AF475286), and *CuPi1* (U93586.1), to silencing and overexpression of *CsTCTP1*/*CsTCTP2* in non-infested cucumber plants by qRT-PCR (**Figure 5**). *CuPi1*, a phloem lectin protein, was more abundant in B21-a-2-2-2 (an *S. fuliginea*-susceptible genotype) than in B21-a-2-1-2 (Meng et al., 2017). The level of *chitinase* transcripts was significantly higher in *CsTCTP1*-silenced plants than in TRV:00-injected plants, whereas it was significantly lower in *CsTCTP1*-overexpressing cucumbers than in LUC:00-injected cucumbers. Additionally, the level of *14-3-3* transcripts was lower in *CsTCTP1*-overexpressing plants than LUC:00-injected plants, and *PR-1a* transcripts were more abundant in *CsTCTP1*-silenced plants than in TRV:00-injected plants. The transcript level of *CuPi1* was higher in *CsTCTP1*-overexpressing plants than LUC:00-injected plants. The *14-3-3, chitinase* and *PR-1a* expression levels were upregulated in *CsTCTP2*-overexpressing plants from their values in LUC:00-injected plants and downregulated in *CsTCTP2*-silenced plants from their levels in TRV:00-injected plants. However, the level of *CuPi1* transcripts exhibited in the opposite trend. These findings indicated that the *CsTCTP1*-induced defense pathway may be linked to defense-associated genes, especially *chitinase*, whereas the *CsTCTP2*-induced defense pathway probably worked in another manner.

### CsTCTP1 and CsTCTP2 Transgenic Plants Modulate the Expression Levels of TOR Signaling- and ABA Signaling-Related Genes

**FIGURE 6:**
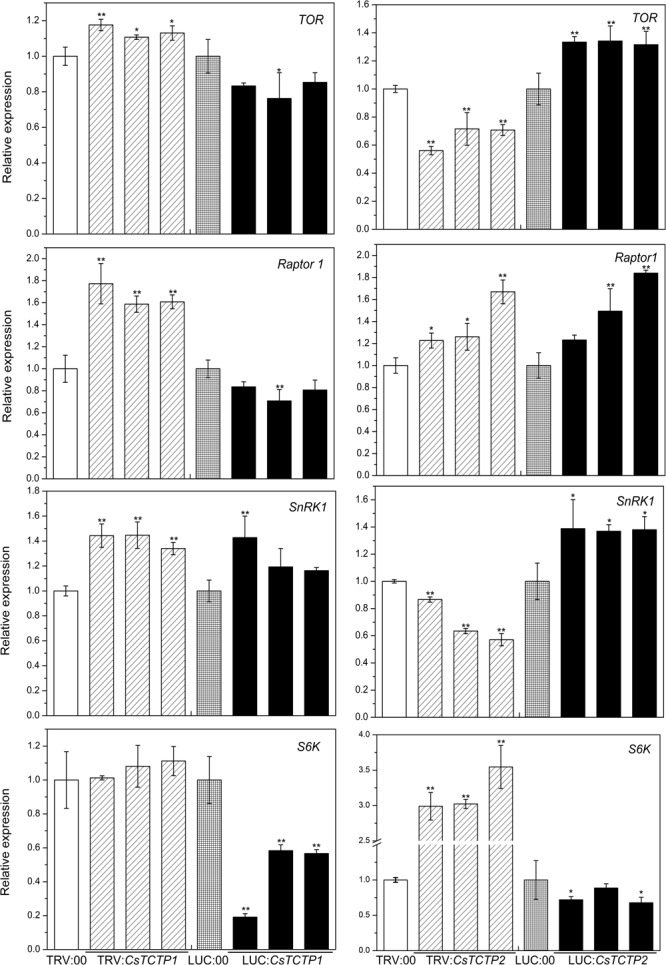
Relative expression levels of four genes (*TOR, SnRK1, S6K*, and *Raptor 1*) related to the TOR signaling pathway in cucumber. The reported transcription levels of the tested genes in the *CsTCTP1*/*CsTCTP2*-silenced and *CsTCTP1*/*CsTCTP2*-overexpressing plants are relative to those in the TRV:00- and LUC:00-injected plants, respectively. Statistically significant differences were identified based on three technical replicates (*t*-test; ^∗^*P* < 0.05, ^∗∗^*P* < 0.01). Bars indicate the standard error of the mean values, and data are presented as the mean values ± SE of three biological replicates.

**FIGURE 7:**
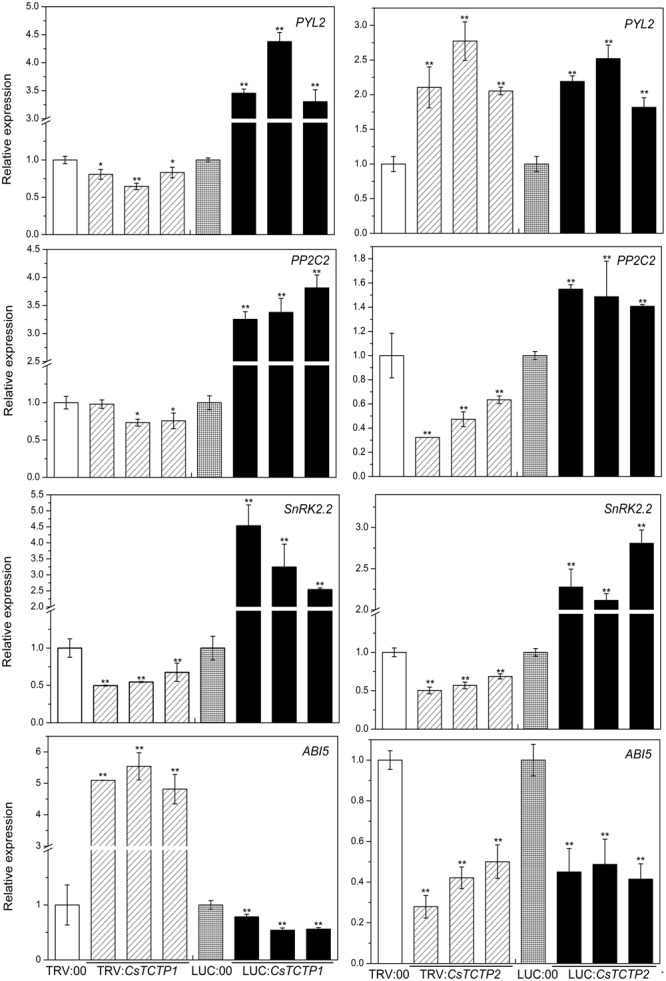
Relative expression levels of four genes (*PYL2, PP2C2, SnRK2.2*, and *ABI5*) related to the ABA signaling pathway in cucumber. The reported transcription of the tested genes in the *CsTCTP1*/*CsTCTP2*-silenced and *CsTCTP1*/*CsTCTP2*-overexpressing plants are relative to those in the TRV:00- and LUC:00-injected plants, respectively. Statistically significant differences were identified based on three technical replicates (*t*-test; ^∗^*P* < 0.05, ^∗∗^*P* < 0.01). Bars indicate the standard error of the mean, and data are presented as the mean ± SE of three biological replicates.

To explore whether CsTCTP1/CsTCTP2 functions in the resistance of transgenic cucumber through the TOR signaling pathway, we conducted the following experiments. *TOR* (XM_011660561.1), *Raptor1* (XM_004149881.2), *S6K* (XM_004138089.2), and *SnRK1* (NM_001305710.1) gene expression was quantified in *CsTCTP1*/*CsTCTP2*-silenced plants and TRV:00-injected plants, as well as in *CsTCTP1*/*CsTCTP2*-overexpressing plants and LUC:00-injected plants (**Figure 6**). *TOR* expression levels exhibited no significant difference between *CsTCTP1*-silenced plants and TRV:00-injected plants, nor does *S6K* between *CsTCTP1*-overexpressing plants and LUC:00-injected plants. The transcript levels of *SnRK1* was higher in both *CsTCTP1*-overexpressing plants and *CsTCTP1*-silenced plants. These results indicated that the expression of *TOR, SnRK1*, and *S6K* may be regulated not only by *CsTCTP1*. The transcript level of *Raptor 1* was at its lowest in *CsTCTP1*-overexpressing plants but was at its highest in *CsTCTP1*-silenced plants. As shown in **Figure 7**, *TOR* and *SnRK1* expression levels were lower in *CsTCTP2*-silenced plants than in TRV:00-injected plants but were higher in *CsTCTP2*-overexpressing plants, and *S6K* performed in the opposite manner. The *Raptor 1* gene was slightly upregulated in both *CsTCTP2*-overexpressing plants and *CsTCTP2*-silenced plants. After treatment with rapamycin (a potential inhibitor to suppress the TOR signaling pathway), cucumbers exhibited remarkably enhanced resistance to *S. fuliginea* (Supplementary Figure S2). These results implied that *CsTCTP2* accumulation is associated with TOR signaling for resistance against *S. fuliginea*.

Abscisic acid is also an important defense signaling molecule. We next examined the possible molecular mode of action of *CsTCTP1* and *CsTCTP2* on the expression of ABA signaling genes (**Figure 7**). The transcript levels of *PYL2* (JF789830), *PP2C2* (JN566067) and *SnRK2.2* (JN566071) genes were significantly higher in the *CsTCTP1*-overexpressing plants than the control plants and lower in the *CsTCTP1*-silenced plants. In contrast, *ABI5* (XM_004149176.2) gene transcription performed in the opposite manner. The relative expression level of the *PYL2* gene was greater in *CsTCTP2*-silenced/overexpressing plants than TRV:00/LUC:00-injected plants, whereas *ABI5* gene expression was downregulated in *CsTCTP2* transgenic plants. The transcript levels of *PP2C2* and *SnRK2.2* genes were higher in *CsTCTP2*-overexpressing plants than LUC:00-injected plants and lower in the *CsTCTP2*-silenced plants than TRV:00-injected plants. These results suggested that *CsTCTP1* positively modulates the expression of *PYL2, PP2C2*, and *SnRK2.2* and negatively modulates the expression of *ABI5*. Moreover, *CsTCTP2* may positively modulate the expression of *PP2C2* and *SnRK2.2*, whereas the expression of *PYL2* and *ABI5* may be regulated not only by *CsTCTP2* but also by other proteins. These data are in line with the ABA content data (Supplementary Figure S3) showing that ABA signaling genes is regulated by *CsTCTP1*.

## Discussion

TCTP is a highly conserved and multifunctional protein in eukaryotic phyla. Increasing amounts of data suggest that TCTP is regulated by pathogen stress. For instance, TaTCTP (*Triticum aestivum*) functions under *Puccinia striiformis* and *Erysiphe graminis* stresses (Li et al., 2010; Wang, 2010). Comparative proteomic studies showed that CsTCTP1 was differentially expressed by two cucumber sister lines (highly resistant and susceptible lines to *S. fuliginea*) subjected to *S. fuliginea* infection (Fan et al., 2014). Here, CsTCTP1 and CsTCTP2 both localized to the cytoplasm, which is in accordance with the *in silico* analysis. This report is the first to determine the subcellular localization of TCTP in cucumber. This study undoubtedly broadens the understanding of the molecular characterization of TCTPs in plant species. Additionally, the different expression patterns of *CsTCTP1* and *CsTCTP2* after pathogen inoculation were associated with the resistance degree in five different cucumber cultivars. We thus deduced that *CsTCTP1* and *CsTCTP2* might related to *S. fuliginea* stress response.

Although TCTP might be involved in responses to *S. fuliginea*, its biological functions remain to be identified. The functional characterization of TCTP in cucumber–pathogen interactions has been hampered until recently by the lack of a gene transformation method in cucumber plants. However, Shang et al. (2014) developed a cucumber cotyledon transient agro-infiltration expression system. In this study, we successfully obtained cucumbers that transiently silenced and overexpressed *CsTCTP1*/*CsTCTP2*. Function analysis revealed that *CsTCTP1*/*CsTCTP2*-silencing markedly enhanced the resistance of these transgenic cucumbers to *S. fuliginea*. In contrast, *CsTCTP1*/*CsTCTP2*-overexpression significantly compromised resistance to *S. fuliginea*. Inoculation with *S. fuliginea* resulted in the typical whitish appearance of PM disease on *CsTCTP1*/*CsTCTP2*-overexpressing cucumber plants and also caused them to exhibit greater hyphal abundances and a higher DI than control plants. These data indicated that both *CsTCTP1* and *CsTCTP2* act as negative modulators in the cucumber defense response to *S. fuliginea*. Notably, overexpression of *CsTCTP1* reduced the tolerance to high temperature stress, while overproduction of *CsTCTP2* enhanced the resistance to high temperature stress (Meng et al., 2017). Since the function of *CsTCTP1* and *CsTCTP2* genes may be similar but do not overlap completely, further study of *CsTCTP1* and *CsTCTP2* regulatory mechanisms is necessary. Studies on TCTP in plants have been conducted by gene transient transformation, mostly in *Arabidopsis* and tobacco. This work is the first to decipher the functions of CsTCTP1 and CsTCTP2 in cucumber defense against *S. fuliginea.* This work undoubtedly provides new insights into TCTP biological functions in cucumber.

According to our results, the cucumber TCTP family gene *CsTCTP1* may activate defense-associated genes in the cucumber defense response to *S. fuliginea*. The transcript level of *chitinase* was more than 20-fold greater in *CsTCTP1*-silenced cucumbers than TRV:00-injected cucumbers, whereas it was significantly lower in *CsTCTP1*-overexpressing cucumbers than LUC:00-injected cucumbers. Another cucumber TCTP family gene, *CsTCTP2*, contributed resistance against *S. fuliginea* but possibly by other means (probably the TOR signaling pathway). The transcript levels of *14-3-3, chitinase*, and *PR-1a* were greater in *CsTCTP2*-overexpressing plants than LUC:00-injected plants and lower in *CsTCTP2*-silencing plants than TRV:00-injected plants. Although CsTCTP1 and CsTCTP2 belong to the same family and are highly similar, they function in the cucumber defense response to pathogens by different mechanisms. Despite having different regulatory mechanisms, the two genes showed the same negative effects on PM.

TOR signaling plays a vital role in balancing cell growth and survival (Aramburu et al., 2014; Hardie, 2015; Schepetilnikov and Ryabova, 2018). Rapamycin is a specific inhibitor of the TOR kinase and produced by *Streptomyces hygroscopicus* (Molnár et al., 1996). However, understanding the mechanisms of TOR signaling has been limited by the absence of FK506 binding protein 12 (FKBP12) in plant cells. Rapamycin can effectively inhibit *Arabidopsis* TOR activity when 10 μM rapamycin is added to liquid cultures of seedlings (Xiong and Sheen, 2014). We applied 10 μM rapamycin to liquid cultures, which resulted in significantly enhanced resistance to *S. fuliginea* and confirmed that the TOR complex negatively regulates pathogen resistance in cucumber (Supplementary Figure S2). Furthermore, diminished expression of the *CsTCTP2* gene in cucumber cotyledons results in increased resistance to *S. fuliginea*. The TORC1 complex, including *TOR* and *Raptor 1*, is regulated by *CsTCTP2*, and a downstream target of TOR signaling, *S6K*, is also regulated by *CsTCTP2*. These results revealed that the response of *CsTCTP2* to *S. fuliginea* was associated with TOR signaling. Extant data indicate that *SnRK1* is crucial for maintaining energy homeostasis in plant cells under stress conditions (Hulsmans et al., 2016; Wurzinger et al., 2018). The transcription level of *SnRK1* was lower here in *CsTCTP2*-silenced plants than TRV:00-injected plants but was higher in *CsTCTP2*-overexpressing plants, suggesting that *CsTCTP2* plays an important role in maintaining energy homeostasis in cucumber under *S. fuliginea* stress.

Abscisic Acid is also an important signaling molecule in plant–pathogen interactions (Audenaert et al., 2002; Sánchez-Vallet et al., 2012; Zhou et al., 2013; Ulferts et al., 2015; Sivakumaran et al., 2016). In this study, *CsTCTP1* positively modulated the expression of *PYL2, PP2C2* and *SnRK2.2* and negatively modulated the expression of *ABI5*. These results revealed that *CsTCTP1* may also be involved in the ABA signaling pathway. The exogenous application of ABA suppresses the resistance of plants to pathogens (Cao et al., 2011). The transcription level of *CsTCTP1* significantly upregulated after treatment with ABA (Meng et al., 2017). Furthermore, augmented expression of the *CsTCTP1* gene in cucumber cotyledons results in decreased resistance to *S. fuliginea.* These results are in accordance with previous evidence showing that ABA levels negatively correlate with resistance to pathogen stress. In a previous study, *ABI5* was expressed more in a resistant line than a susceptible line after *S. fuliginea* infestation (Chen, 2017). In the current study, the silencing of *CsTCTP1* in cucumber cotyledons upregulated *ABI5* transcription and simultaneously enhanced resistance to *S. fuliginea*. These results are also consistent with the response of *CsTCTP1* to *S. fuliginea* being associated with the ABA signaling pathway.

## Conclusion

*CsTCTP1* and *CsTCTP2* are negative modulators in the cucumber defense response to *S. fuliginea* stress. *CsTCTP1* participates in the defense response to *S. fuliginea* through regulating the expression of certain defense-associated genes and/or ABA signaling pathway-associated genes, and *CsTCTP2* participates by regulating the expression of TOR signaling pathway-associated genes. Our findings provide new insights into TCTP function in cucumber defense responses to PM and will guide enhancing the resistance of cucumber to pathogens.

## Author Contributions

HF conceived and designed the research. XM analyzed the data and wrote the manuscript. XM, YaY, JZ, NC, TS, and YuY conducted the experiments. All authors read and approved the manuscript.

## Conflict of Interest Statement

The authors declare that the research was conducted in the absence of any commercial or financial relationships that could be construed as a potential conflict of interest.
