# Supplementary material for: The Two Translationally Controlled Tumor Protein Genes, CsTCTP1 and CsTCTP2, Are Negative Modulators in the Cucumis sativus Defense Response to Sphaerotheca fuliginea
Source: Front Plant Sci. 2018 Apr 25;9:544. doi: 10.3389/fpls.2018.00544 (PMC5996911; doi:10.3389/fpls.2018.00544)
Supplement: Supplementary file 1 [file Image_1.pdf]

Supplementary Material

The two translationally controlled tumor protein genes, *CsTCTP1* and *CsTCTP2*, are negative modulators in the *Cucumis sativus* defense response to *Sphaerotheca fuliginea*

Xiangnan Meng[1, 2], Yang Yu[1], Junyue Zhao[1], Na Cui[1], Tiefeng Song[4], Yun Yang[1] and Haiyan Fan[1, 3*]

[1] College of Bioscience and Biotechnology, Shenyang Agricultural University, Shenyang, China

[2] College of Horticulture, Shenyang Agricultural University, Shenyang, China

[3] Vegetable Research Institute, Liaoning Academy of Agricultural Sciences, Shenyang, China

[4] Key Laboratory of Protected Horticulture of Ministry of Education, Shenyang Agricultural University, Shenyang, China

Correspondence[*]:
Prof. Haiyan Fan
Hyfan74@163.com

1 Supplementary Table

Name	Primer
GFP1	F: 5'-GCTCTAGAGCATGTTGGTTTATCAGGAC-3'<br>R: 5'-CGAGCTCGTGCACTTGACTTCCTT-3'
GFP2	F: 5'-GCTCTAGAGCATGCTTCTCTACCAAGAC-3'<br>R: 5'-CGAGCTCGGACACTTGACTTCCTTCAAC-3'
1-RT	F: 5'-GGACAAGAAGGTTTTCCTCACA-3'<br>R: 5'-GGAACTTAACTGCTCCCTCAAT-3'
2-RT	F: 5'-AGACGAAGAAAAGCAAGAGTTG-3'<br>R: 5'-TCCCTTCACCCACAAAGAAT-3'
LUC1	F: 5'-TCCAGATCTGAGCTCTCTAGAATGTTGGTTTATCAGGAC-3'<br>R: 5'-GTCTTCCATAAGCTTCTGCAG GCACTTGACTTCCTT-3'
LUC2	F: 5'-TCCAGATCTGAGCTCTCTAGAATGCTTCTCTACCAAGAC-3'

	R: 5'-GTCTTCCATAAGCTTCTGCAGACACTTGACTTCCTTCAAC-3'
TRV1	F: 5'-GCTCTAGAGCTTGCTCTCCTTTCAGCAAAG-3'
	R: 5'-CGAGCTCGATGTTGGTTTATCAGGACCTC-3'
TRV2	F: 5'-GCTCTAGAGCCTTTTCTTCGTCTAGTTTAG-3'
	R:5'-CGAGCTCGATGCTTCTCTACCAAGAC-3'
TCTP1	F: 5’-GGAGCAGTTAAGTTCCTACTTCCAAAGG -3’
TCTP2	F: 5’-CCAGAGGCAACCAAGTTTCTCATTTCG -3’
LUC	F: 5’-CAGCGTAAGTGATGTCCACCTCG -3’
Actin	F: 5'-TGCTGGATTCTGGTGATGGTGTGA-3'
	R: 5'-AGGTCCAAACGGAGAATGGCATGA-3'
14-3-3	F: 5'-TCCACTAAAGAACGCGACAACTTC-3'
	R: 5'-CCTTTTGTCTGTATTCTTTGATGCG-3'
Chitinase	F: 5'-GCCGCAGTGTCCAATACCAG-3'
	R: 5'-TCAGGAGATTGTCCGCGTTA-3'
PR-1a	F: 5'-GAACTCTGGCGGACCTTA-3'
	R: 5'-GCATCTCACTTTGGCACATC-3'
CuPi1	F:5'-GCACCAAAACAACGAAAAGG-3'
	R:5'-GGCTATAAGGACCGCTACCAT-3'
TOR	F: 5'-ACGGCTAAGGCATCACAAGCATC-3'
	R: 5'-AAGGCATCTCATCCGCCCTAAC-3'
SnRK1	F: 5'-AAGTGGGCTTTGGGATTACAGTC-3'
	R: 5'-AGCCACATTAAGCTCCCGTAGG-3'
Raptor1	F: 5'-CGCTTTAGCCAATGACCCA-3'
	R: 5'-AAGGCAAATGACCTCCGTTC-3'
S6K	F: 5'-AGCTTCGCTACTCTTTCCAGACC-3'
	R: 5'-GCTGCATAGACTCTTGCCTGATCC-3'
PYL2	F: 5'- CACCACAACCACAATCCCACAC-3'
	R: 5'-TCCTGACCAACGACCATACGAG-3'
PP2C2	F: 5'-TCGGTCGATCATAAACCAAATCGC-3'
	R:5'-ACGATGTCCGTTCCACTGGATG-3'
SnRK2.2	F: 5'-TCAGGAGTCAGCTACTGCCATTC-3'
	R: 5'-GGGAATGCAACACTGATGACTTGG-3'
ABI5	F: 5'-TTTGGGTTGGACTTGGGTG-3'
	R:5'-CTTTTCCTTTGCTCTTTGTGCT-3'

2 Supplementary Figures

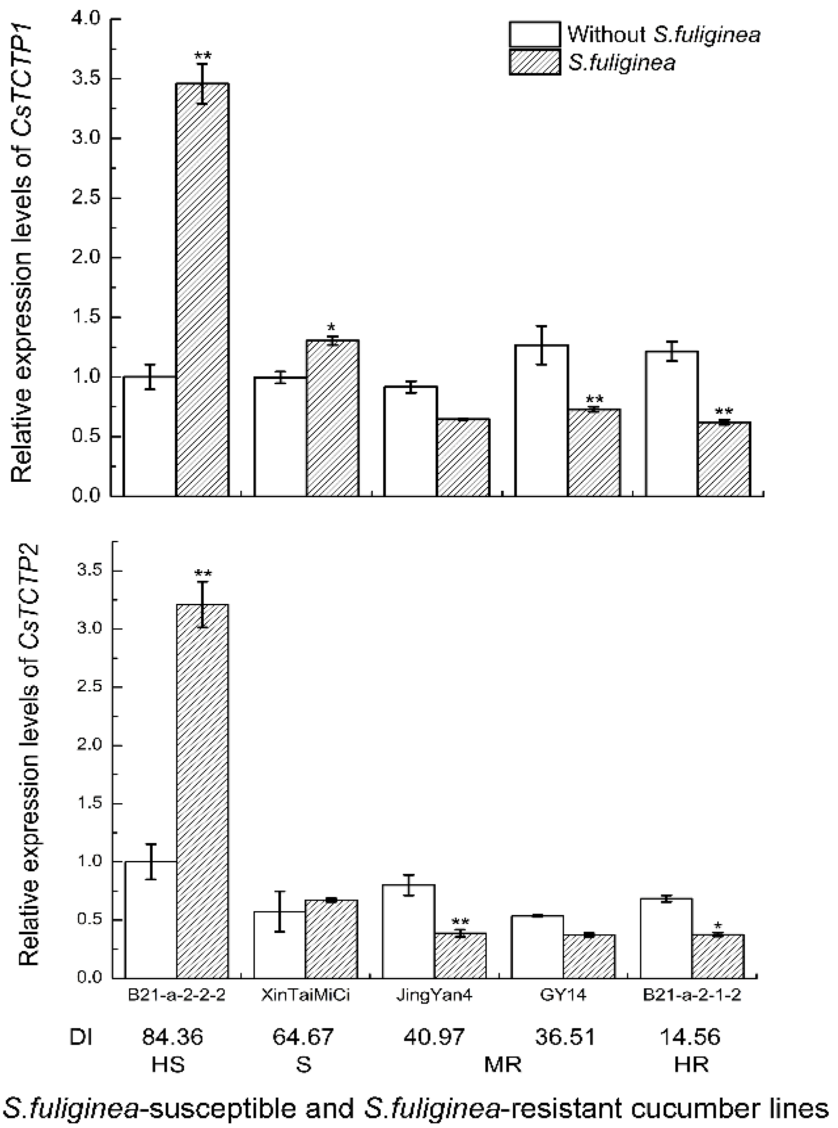

Figure S1 Expression patterns of *CsTCTP1* and *CsTCTP2* in five cucumber cultivars with different degrees of resistance to *S. fuliginea*. The expression levels of *CsTCTP1* and *CsTCTP2* in the B21-a-2-2-2 (highly susceptible) line without pathogen for 7 days were set to 1. Data are presented as the means ± SEs of three biological replicates. Letters indicate significant differences at $P < 0.05$ by Student's t-test. HS indicates highly susceptible, S indicates susceptible, MR indicates moderately resistant, HR indicates highly resistant, and DI indicates the disease index of powdery mildew.

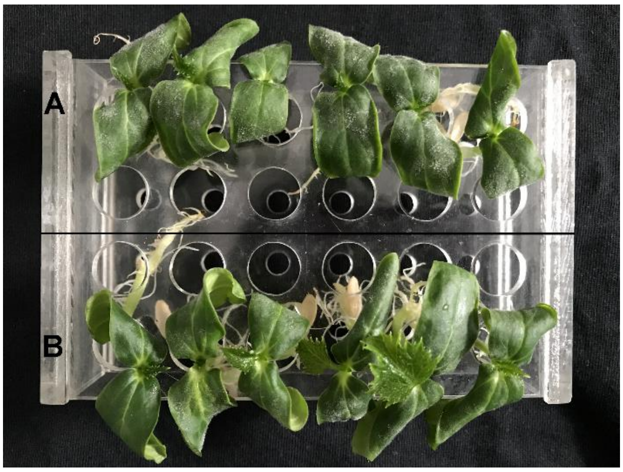

Figure S2 Disease symptoms of the control and the rapamycin-treated cucumbers at 7 dpi with *S. fuliginea*. (A) Control cucumbers and (B) rapamycin-treated cucumbers.

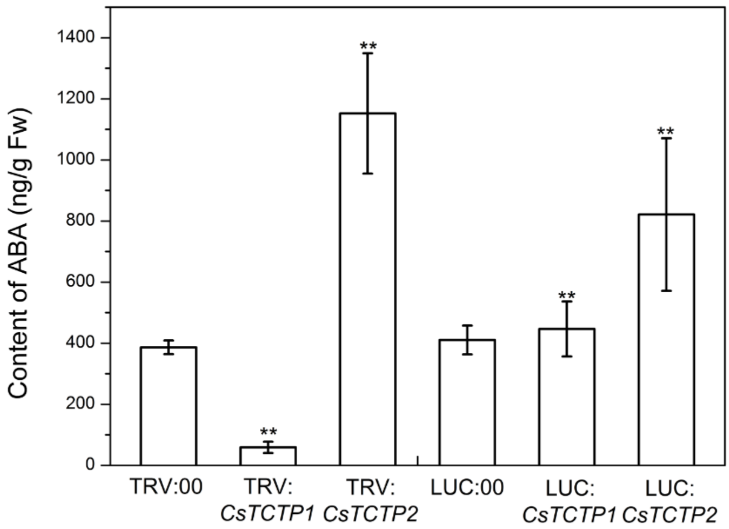

Figure S3 ABA content in TRV:00-injected, TRV:*CsTCTP1*-injected, TRV:*CsTCTP2*-injected, LUC:00-injected, LUC:*CsTCTP1*-injected, and LUC:*CsTCTP2*-injected cucumbers. Data are presented as the means ± SEs of three biological replicates. Letters indicate significant differences at $P < 0.05$ by Student's t-test.
